# Incidence of unlicensed and off-label prescription in children

**DOI:** 10.1186/1824-7288-40-12

**Published:** 2014-02-04

**Authors:** Petra Langerová, Jiří Vrtal, Karel Urbánek

**Affiliations:** 1 Department of Pharmacology, Faculty of Medicine and Dentistry, Palacky University and University Hospital Olomouc, Hněvotínská 3, 77515 Olomouc, Czech Republic

**Keywords:** Prescribing habits, Off-label drug use, Pediatric outpatient clinics

## Abstract

**Background:**

Many common drugs have not been licensed for use in children.

**Methods:**

This study evaluated the incidence of unlicensed and off-label prescriptions at the Department of Pediatrics during a period of six months. A total of 8,559 prescriptions for 4,282 children were processed.

**Results:**

Off-label and unlicensed prescriptions were found in 9.01% and 1.26% of all prescriptions, respectively. Unlicensed prescriptions were significantly more common in boys (1.5%) than in girls (1.0%) (p = 0.037). There was no significant difference between off-label prescriptions in boys (9.0%) and in girls (9.1%) (p = 0.89). The prescription of unlicensed drugs was significantly more frequent in school age children (p < 0.0001). The most commonly prescribed unlicensed drugs were angiotensin-converting enzyme inhibitors; among off-label drugs, antihistamines and bronchodilators.

**Conclusions:**

This study shows that the incidence of unlicensed and off-label drug prescriptions in our patients is not as high as in other studies.

## Introduction

Many common drugs used to treat children are either not licensed for use in children at all (*unlicensed drugs*) or are prescribed outside the terms of the product licence (*off-label prescription*) [1]. Drugs can be prescribed off-label for younger children, in a different dose, for a different indication or by a different route of administration than which the drug is licensed for. The use of unlicensed and off-label medicines in children is common because clinical trials have not usually been performed in children during the research and development process. Consequently, the information available to pediatricians may not always be as detailed or as robust as when prescribing a medicine that is licensed for an approved indication. This has led to concerns that children can receive drugs at dosages that either lack efficacy or exhibit safety problems. The latter in particular has received a great deal of attention [2,3].

Childhood diseases may be different from their adult equivalents. This may affect either the benefit and/or the risk of therapy. The lack of reliable data in the pediatric population is associated with specific problems, including limited availability of safety data due to the lack of clinical trials and insufficient pharmacokinetic data or dose-finding studies. Maturation, growth and development make the pediatric population susceptible to drug-induced growth and development disorders as well as to delayed adverse drug reactions (ADRs). Pediatricians should be aware that the use of drugs prescribed off-label may increase the risk of adverse reactions [4].

Based on this, there was a need for a legal obligation for pharmaceutical companies to perform studies if they intended to develop medicines for use in the pediatric population. In 1997, the European Commission discussed problematic issues of pediatric medicines. One of the conclusions was that there was a need to strengthen the legislation. In 1998, the Commission supported the need for international discussion on the performance of clinical trials in children in the context of the International Conference on Harmonization (ICH) – an organization working on the harmonization of pharmaceutical regulatory requirements between the EU, Japan and the US. An ICH guideline was therefore adopted. The goals were to encourage and facilitate timely pediatric medicinal product development internationally, and to provide an outline of critical issues in pediatric drug development and approaches to a safe, efficient and ethical study of medicinal products. Subsequently, the ICH guideline became the European guideline. The Directive on Good Clinical Practice for Clinical Trials came fully into force in 2004. This Directive takes into account some specific concerns about performing clinical trials in children, and it lays down criteria for their protection in clinical trials [5].

Moreover, off-label use of medicines in children is still common. As reported by Santos et al., off-label drug use affects 36%–92% of hospitalized children [6]. Therefore, we intended to evaluate the current incidence of unlicensed and off-label prescriptions in outpatients during a period of six months to identify the drugs requiring an extension of registration for younger children.

## Methods

### Study design and setting, study population

Prescription data of all outpatients aged 0–15 years attending the University Hospital Olomouc, Czech Republic, during a 6-month period (from 1^st^ January to 30^th^ June 2012) were processed. Patients who reached 15 years of age in the follow-up period were included in the study population. Patients older than 15 years did not enter this study. The following characteristics were recorded for each patient: age, sex, and the number of prescriptions. In addition, the incidence of unlicensed and off-label prescription of drugs was recorded and the drugs most commonly prescribed were identified.

### Consent

The study was conducted following the guidelines of the Declaration of Helsinki. Written informed consent to participate was not required because this was an observational study. During the study no medical interventions were done.

### Ethical approval

All ethical considerations were followed. Patient files were processed anonymously. No personal data were collected. The research project was approved by FN and LF UP Olomouc Institutional Ethical/Review Board.

### Data processing

Terms of the license were obtained from the Summary of Product Characteristics. The drugs that were not explicitly licensed for use in children were considered as unlicensed. The off-label category included all medicines where the prescription showed a discrepancy with the licence information for age; i.e. if the drug was prescribed for a younger child than for which age it was licensed.

### Statistical analysis

For all the data analyses, Pearson’s chi-square test was used. It tested a null hypothesis stating that the frequency distribution of certain events observed in a sample was consistent with a particular theoretical distribution. The events considered had to be mutually exclusive and had to have total probability. Confidence intervals were stated at the 95% level.

## Results

A total of 4,282 outpatients younger than 15 years of age entered the study. During the 6-month follow-up period, they received 8,559 prescriptions. The descriptive statistics of the study cohort are given in Table 1. Unlicensed drugs were prescribed in 1.26% (95% CI 1.02–1.50); off-label drugs were prescribed in 9.01% (95% CI 8.40–9.62) of all prescriptions. The distribution of unlicensed and off-label drug use in different age categories is shown in Table 2.

**Table 1:** Distribution of the number of patients and prescriptions in different age categories

***Age category***	***Age***	***Patients***	***Females***	***Prescriptions in females***	***Males***	***Prescriptions in males***	***Prescriptions total***	***Total prescriptions per patient***
**Newborns**	0–1 m	18	5	5	13	17	22	1.22
**Infants**	1 m–1 yr	304	133	214	168	283	497	1.63
**Toddlers**	1–3 yrs	609	308	515	301	514	1029	1.69
**Preschoolers**	3–6 yrs	1027	626	1249	401	774	2023	1.97
**Early school age children**	6–11 yrs	1296	763	1516	533	1164	2680	2.07
**School age children**	11–15 yrs	1028	465	1076	563	1232	2308	2.25
**Total**	**0–15 yrs**	**4282**	**2300**	**4575**	**1979**	**3984**	**8559**	**2.00**

**Table 2:** **Distribution of unlicensed and off-label prescriptions in different age categorie**s

	***Prescriptions total***	***Unlicensed***	***Off-label***
**Newborns**	22	0	9
**Infants**	497	1	71
**Toddlers**	1029	0	134
**Preschoolers**	2023	16	309
**Early school age children**	2680	29	156
**School age children**	2308	62	93
**Total**	**8559**	**108**	**772**

Drugs prescribed unlicensed were significantly more common in boys (1.5%, 95% CI 1.15–1.91) than in girls (1.0%, 95% CI 0.74–1.32) (p = 0.037). There was no significant difference between off-label prescription in boys (9.0%, 95% CI 8.08–9.85) and in girls (9.1%, 95% CI 8.22–9.88) (p = 0.89). See Table 3.

**Table 3:** Gender-related distribution of unlicensed and off-label prescriptions

	***Total***	***Unlicensed***	***Off-label***
**Males**	3984	61	358
**Females**	4575	47	414
**Total**	8559	108	772

Unlicensed use of drugs was significantly more frequent in school age children than in any other age categories (p < 0.0001). Unlicensed drugs were prescribed in 2.7% (95% CI 2.07–3.40) of school age children (11–15 years), in 1.0% (95% CI 0.69–1.47) of early school age children (7–11 years) and in less than 1.0% in other age categories. There has been a significant difference in the frequency of off-label drug use between individual age categories (p < 0.0001). Drugs were most frequently prescribed off-label for newborns (40.9%, 95% CI 20.36–61.45), but could not be evaluated because of a low number of patients of this age in our study. Drugs prescribed off-label were more frequently found in infants (14.3%, 95% CI 11.21–17.36), toddlers (13.0%, 95% CI 10.97–15.08) and preschool age children (15.3%, 95% CI 13.72–16.86).

The individual drugs most commonly prescribed unlicensed and off-label are presented in Tables 4 and 5. The most commonly prescribed unlicensed drugs were angiotensin-converting enzyme (ACE) inhibitors (ramipril and enalapril). The prescription of ramipril occurred in 24 prescriptions. Of this total, 17 prescriptions were for boys (in 12 individuals) and 7 for girls (4 individuals). Among off-label drugs, antihistamines (desloratadine and cetirizine) and bronchodilators were the most common.

**Table 4:** Drugs most commonly prescribed unlicensed

***Generic name***	***Trade name***	***Prescriptions***	***Patients***
Porcine transfer factor	Imunor por. lyo. 4 × 10 mg	6	5
Cyproterone	Androcur-50 tbl. 50 × 50 mg	5	5
Ramipril	Tritace tbl. 50 × 1,25 mg	5	5
Enalapril	Apo-enalapril tbl. 100 × 5 mg	4	4
Ramipril	Tritace tbl. 50 × 5 mg	3	3
Ramipril	Tritace tbl. 30 × 2,5 mg	3	3
Ramipril	Amprilan tbl. 30 × 2,5 mg	3	2
Metoclopramide	Degan tbl. 40 × 10 mg	3	3
Atenolol	Tenormin tbl. 28 × 50 mg	3	2
Ramipril	Tritace tbl. 50 × 2,5 mg	2	2

(Drugs licensed for adults only).

por. lyo. – oral lyophilisate.

tbl. – tablet.

**Table 5:** Drugs most commonly prescribed off-label

***Generic name***	***Trade name***	***Prescriptions***	***Patients***	***Approved for patients older than (years)***
Salbutamol	Ventolin inhaler N 200 × 100RG	43	42	4
Desloratadine	Aerius tbl.dis. 90 × 2,5 mg	48	47	6
Mometasone	Nasonex nasal spray 140 × 50RG	32	30	6
Desloratadine	Aerius tbl. 90 × 5 mg	34	33	12
Cetirizine	Zodac gtt. 1 × 20 ml	28	28	2
Clarithromycin	Klacid tbl. 14 × 250 mg	28	26	12
Fluticasone furoate	Avamys nasal spray 120 × 27,5RG	25	22	6
Codeine	Codein Slovakofarma tbl. 10 × 15 mg	14	13	12
Promethazine	Prothazin tbl. 20 × 25 mg	14	14	10
Montelukast	Singulair 5 Junior tbl.mnd. 98 × 5 mg	14	14	6
Phenoxymethylpenicillin	Ospen 500 tbl. 30 × 500KU	17	17	6
Desloratadine	Aerius tbl. 50 × 5 mg	14	14	12

tbl. – tablet.

tbl. dis. – orodispersible tablet.

tbl. mnd. – chewable tablet.

## Discussion

Previous studies have shown that unlicensed and off-label drug use in children is common. As reported by Pandolfini and Bonati, off-label prescriptions in pediatric studies in the community affected 9%–33% of children [7].

Pandolfini and Bonati performed a literature review of all published studies on off-label and unlicensed drug use in children. A total of 30 studies from 1985 to 2004 were included. Eleven involved pediatric hospital wards, seven neonatal hospital wards, and twelve the community setting. The off-label and unlicensed classification methods varied, making the results difficult to compare. In general, off-label/unlicensed prescription rates ranged from 11% to 80%, and higher rates were found in younger versus older patients and in the hospital versus community settings. In the pediatric hospital wards, off-label/unlicensed prescriptions ranged from 16% to 62% and most often involved acetaminophen, cisapride, chloral hydrate and salbutamol. In the community setting, rates ranged from 11% to 37% and the most commonly prescribed unlicensed or off-label drugs were salbutamol and amoxicillin [7].

More recently, an interesting review of this area of interest was also published by Lindell-Osuagwu et al. [8]. However, the definition of unlicensed and off-label drug use varied among different studies. Therefore, the results are not directly comparable but provide an overall picture of the issue [8].

Unlike these earlier studies, our results show that the current incidence of unlicensed and off-label prescriptions in our outpatients is not as high as has been previously found. Drugs prescribed off-label were found in 9.01%; unlicensed drugs were identified in only 1.26% of all prescriptions.

The most frequently used drugs were searched for in a relatively recent study by Hsian et al. [9]. They conducted a prospective observational study at a pediatric ward in Germany between January and June 2006. The study included 417 patients for whom 1,812 prescriptions representing 211 different drugs were made. Unfortunately, in this study the term “off-label” was used for both unlicensed and off-label prescriptions. In total, 253 patients (61%) received at least one off-label prescription; 553 (31%) of all prescriptions were off-label. The five drug groups most frequently prescribed off-label were: cardiovascular drugs (60% of prescriptions), anti-infectives (42%), drugs of the respiratory system (30%), drugs of the alimentary tract and metabolism (25%) and analgesics and antipyretics (3%). The cardiovascular drugs also exhibited the highest number of drugs prescribed off-label due to the patient’s age [9].

In our study, the cardiovascular drugs (mainly ACE inhibitors and beta blockers) were also among the most frequently prescribed unlicensed drugs. Their use is sometimes necessary, because no effective licensed alternatives are available. This is mainly the case of captopril or enalapril used in children with hypertension or heart failure. This finding highlights the necessity of registering cardiovascular drugs for younger children as well.

Recent studies also show a relationship between off-label and unlicensed drug prescribing and age. This prescription was repeatedly found to be most common in newborns. The younger the child, the more likely he/she is to get an off-label or unlicensed drug prescription. This was found mainly in the hospital care. On the contrary, in outpatient settings, a higher rate of these prescriptions has been found in adolescents. There is only a slight difference between individual countries where studies were performed [8,9].

The results of our study were influenced by number of patients in each age category. The prescription of unlicensed drugs was significantly more frequent in school age children than in any other age group. In this category, there is also the highest number of prescriptions per patient. It is probably caused by the morbidity of children. The older the child, the higher is the risk of multiple therapies due to chronic diseases. Off-label prescriptions were most frequent in newborns, but the low number of patients in this age category prevented making any definite conclusions.

Drugs prescribed unlicensed were significantly more common in boys than in girls, but there was no such gender-related difference in off-label prescription. This difference was found to be caused by several unlicensed cardiovascular drugs, which were prescribed in boys more often.

It is surprising that only very few recent studies have evaluated the link between gender and unlicensed or off-label drug prescribing. One of them is a study by Santos et al. No statistically significant differences between boys and girls were found, however with some exceptions: the prevalence of corticosteroids for systemic use was higher in females than males (among children under 1 year only), and the same was observed for anti-asthmatics among 2–5-year-old children and for diuretics in children under 6 years of age [6].

In our study, more boys were treated with ACE inhibitors. The main reason is that ramipril is not licensed for children and it was substantially more commonly prescribed in boys (M/F ratio 12:4). This might be influenced by a higher incidence of cardiovascular diseases in boys. The most common indication for ramipril was hypertension, particularly caused by renal impairment. In the majority of cases, the hypertension in boys was caused by polycystic kidney disease (PKD), an autosomal recessive disease, a significant cause of renal and liver-related morbidity in children and adolescents [10]. However, no relationship between sex and PKD has been described [11].

More probable explanations are offered in a recent study that discovered that boys had significantly higher values for height, body weight, waist circumference, BMI and systolic blood pressure than girls. Diastolic blood pressure tends to be higher in boys than in girls [12]. This can explain the intersexual difference in ACE inhibitors use found in our study, but further research into this topic is needed.

The majority of drugs prescribed off-label were antihistamines and drugs used to treat respiratory diseases. These drugs are usually licensed for children older than 6 years. The incidence of allergic and respiratory diseases in preschool children is very high [13], therefore antihistamines belong to the most frequently prescribed drugs for them. The first generation of antihistamines has never been clinically tested in children; nevertheless, it is widely used [14]. Therefore, clinical trials in younger children and extension of registration also for younger patients are needed.

Salbutamol in the form of pressurized suspension for inhalation was the individual active agent most frequently prescribed off-label. This result is consistent with those of several other studies [15-18]. Salbutamol has only been approved for children older than 4 years, i.e. its prescription for younger patients is off-label. For younger children, only liquid drug formulation (salbutamol syrup) is available. However, the use of inhalation drug form is often more suitable. Thus, an extension of its licence would also be advisable.

## Conclusions

Unlicensed and off-label prescribing in children is common and widespread. The problem has been recognized by many researchers [18-21]. Our goal was to determine the extent of use of drugs that are not specifically licensed for use in children (unlicensed) and of those used outside the terms of their licence (off-label) with respect to the age of the child. This study shows that the incidence of unlicensed and off-label drug prescriptions in our patients is not as high as in other studies. Drugs prescribed off-label and unlicensed were found in 9.01% and 1.26% of all prescriptions, respectively. Although the quality of drug therapy is not necessarily related to drug license status, pediatricians should avoid exposing children to unnecessary risks and depriving them of potentially effective pharmacotherapy. To achieve this goal, an amendment of legislation to facilitate and encourage pediatric clinical trials is in process. The results of our study could guide the researchers to perform future trials mainly on cardiovascular drugs, antihistamines and respiratory system drugs.

## Competing interests

The authors declare that they have no competing interests.

## Authors’ contributions

Authors PL and JV recorded the following characteristics for each patient: age, sex, and the number of prescriptions. In addition, the incidence of unlicensed and off-label prescription of drugs was recorded and the drugs most commonly prescribed were identified. Author PL as well created all tables and drafted the manuscript. Author KU revised the results and the manuscript. All authors read and approved the final manuscript.
